# Case report: A toxoplasmic encephalitis in an immunocompromised child detected through metagenomic next-generation sequencing

**DOI:** 10.3389/fpubh.2023.1247233

**Published:** 2023-09-28

**Authors:** Chuang-Wei Yu, Xiong-Feng Zhu, Chongjian Huang, Hua-Dong Meng, Xiao-Guang Cao

**Affiliations:** ^1^Department of Emergency Intensive Care Unit, TaiHe County People’s Hospital, Fuyan, China; ^2^Department of Emergency Emergency Internal Medicine Department, The Third People's Hospital of Hefei, Hefei, China; ^3^Department of Emergency Intensive Care Unit, The First Affiliated Hospital of University of Science and Technology of China (Anhui Provincial Hospital), Hefei, China; ^4^Department of Emergency Intensive Care Unit, The Third Affiliated Hospital of AnhuiMedical University (The First People's Hospital of Hefei), Hefei, China

**Keywords:** mNGS, toxoplasmic encephalitis, CNS infections, diagnostic tool, immune dysfunction

## Abstract

There exist numerous pathogens that are capable of causing infections within the central nervous system (CNS); however, conventional detection and analysis methods prove to be challenging. Clinical diagnosis of CNS infections often depends on clinical characteristics, cerebrospinal fluid (CSF) analysis, imaging, and molecular detection assays. Unfortunately, these methods can be both insensitive and time consuming, which can lead to missed diagnoses and catastrophic outcomes, especially in the case of infrequent diseases. Despite the application of appropriate prophylactic regimens and evidence-based antimicrobial agents, CNS infections continue to result in significant morbidity and mortality in hospital settings.

Metagenomic next-generation sequencing (mNGS) is a novel tool that enables the identification of thousands of pathogens in a target-independent manner in a single run. The role of this innovative detection method in clinical pathogen diagnostics has matured over time. In this particular research, clinicians employed mNGS to investigate a suspected CNS infection in a child with leukemia, and unexpectedly detected Toxoplasma gondii.

**Case:**

A 3-year-old child diagnosed with T-cell lymphoblastic lymphoma was admitted to our hospital due to a 2-day history of fever and headache, along with 1 day of altered consciousness. Upon admission, the patient’s Glasgow Coma Scale score was 14. Brain magnetic resonance imaging revealed multiple abnormal signals. Due to the patient’s atypical clinical symptoms and laboratory test results, determining the etiology and treatment plan was difficulty.

Subsequently, the patient underwent next-generation sequencing examination of cerebrospinal fluid. The following day, the results indicated the presence of Toxoplasma gondii. The patient received treatment with a combination of sulfamethoxazole (SMZ) and azithromycin. After approximately 7 days, the patient’s symptoms significantly improved, and they were discharged from the hospital with oral medication to continue at home. A follow-up polymerase chain reaction (PCR) testing after about 6 weeks revealed the absence of Toxoplasma.

**Conclusion:**

This case highlights the potential of mNGS as an effective method for detecting toxoplasmic encephalitis (TE). Since mNGS can identify thousands of pathogens in a single run, it may be a promising detection method for investigating the causative pathogens of central nervous system infections with atypical features.

## Introduction

In recent years, CNS infections have become increasingly prevalent worldwide neurological disorders related to CNS infections are common in China (1–6). Despite the availability of appropriate prophylactic regimens and evidence-based antimicrobials, these infections continue to cause significant morbidity and mortality in hospitals, particularly among immunocompromised patients (7, 8). They account for a significant proportion of functional disability and financial burden.

However, identifying the pathogens in CNS infections using conventional methods such as culture, PCR, and serological tests may be laborious (9, 10). In addition, atypical symptoms and a lack of knowledge can further increase diagnostic difficulties. Consequently, identifying the pathogen using conventional methods seems to be more laborious and inefficient in clinical practice, especially for rare pathogens such as Toxoplasma gondii (11, 12). mNGS is a highly advanced technology that allows for rapid sequencing of large amounts of DNA or RNA. In recent years, mNGS has been increasingly used in clinical diagnostics and research, including in the detection of infectious diseases (13–15).

TE is a severe condition caused by the parasite Toxoplasma gondii (16). It can affect individuals with weakened immune systems, such as those with HIV/AIDS, leukemia patients, and even pregnant women and newborns. The diagnosis of TE can be more difficult than other CNS infections because the symptoms may be non-specific and rare, and the condition can mimic other diseases, especially in patients with immune disorders, such as children with leukemia (17). Recent studies have investigated the value of mNGS in the diagnosis of TE (12, 18). These studies have shown that mNGS can effectively detect T. gondii DNA in cerebrospinal fluid samples from patients with suspected TE, with high sensitivity and specificity.

## Case

**Figure 1:**
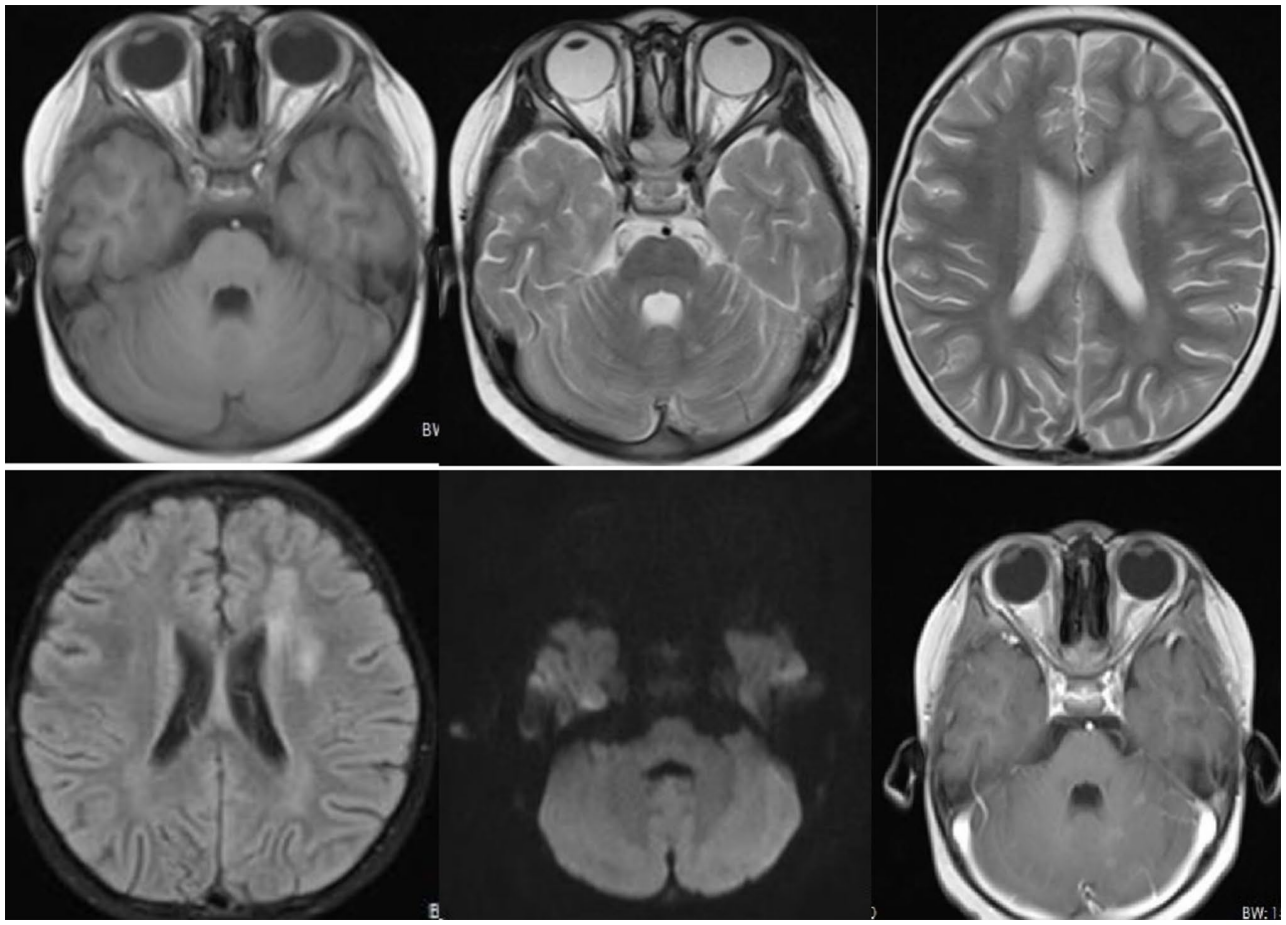
Radiological findings revealed patchy and slightly longer T1 and T2 signals with unclear boundaries and high signal intensity on T2-weighted fluid-attenuated inversion recovery (T2WI-FLAIR) in the right parietal lobe, left temporal lobe, and left lateral ventricular forefoot. The bilateral hippocampi demonstrated patchy, slightly longer T1 and T2 signals with unclear boundaries and high signal on T2WI-FLAIR. The left cerebellar hemisphere displayed multiple patchy and slightly longer T1 and T2 signals with clear boundaries and patchy enhancement on the contrast-enhanced scan. Long linear T1 and T2 signals were observed in the right parietal lobe, with linear enhancement seen on the contrast-enhanced scan.

In October 2020, a 3-year-old patient was diagnosed with T-cell lymphoblastic lymphoma and received chemotherapy, although the specific drugs used are unknown. On April 29th, 2021, the patient was admitted to our hospital’s Hematopoietic Stem Cell Transplantation Department. During their hospitalization, they underwent a conditioning regimen that included Carmustine, Fludarabine, Busulfan, and Cyclophosphamide. Simultaneously, the patient’s pre-transplant examination results were in alignment with the transplant criteria. Subsequent to this, on May 7th, 2021, the patient underwent a 25 mL cord blood stem cell treatment and was discharged 1 month later. However, on July 22nd, the patient’s condition deteriorated without an obvious cause, and 2 days ago, the patient developed a fever with a peak temperature of 38°C. Consequently, the patient was referred to the outpatient department for treatment with Azithromycin, Paracetamol, and Oseltamivir. Nevertheless, the symptoms did not improve, and the patient was admitted to the inpatient department for further evaluation and treatment because of the severity of the symptoms and unclear diagnosis. Upon arrival, the patient had a blood pressure of 126/73 mmHg, a heart rate of 154 bpm, and a respiratory rate of 38 times/min. The patient was afebrile (37.2°C). Physical examination revealed weakness, headache, and lethargy, but did not demonstrate any signs of a stiff neck, mental disorders, movement disorder, speech disorder, or any other abnormal nerve examination. As the cause of the illness was not apparent, the patient was given a combination of Ganciclovir, Vancomycin, and Carbapenems. The doctors collected various samples for testing, including sputum, urine, blood, and cerebrospinal fluid; these routine test results and magnetic resonance imaging (Figure 1) did not provide very valuable information (Table 1). However, during this period, the patient’s symptoms did not demonstrate significant improvement.

**Table 1:** Cerebrospinal fluid test.

Open Pressure		92 mmHg
Colors		Transparent
Cell Count	Karyocyte cell	5 × 10^6/L
	Red blood cell	0
	Mononuclear cell	100%
	Multinucleated cell	0%
Glucose		3.55 mmol/L
Protein		0.42 g/L
Chloride		120.0 mmol/L

**Figure 2:**
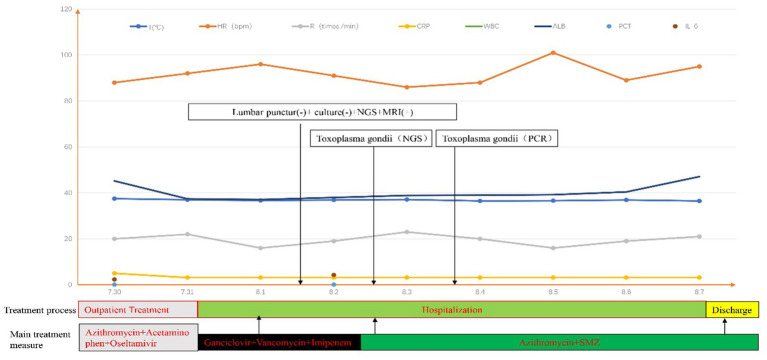
“+” means positive; “-” means negative. The beginning and end of each color bar showed the primary treatment measures during this period.

Meanwhile, the patient’s cerebrospinal fluid sample underwent pathogenic microorganism analysis using the BGI PMSeq high-throughput sequencing process. 1.5-3 mL CSF from patient was collected according to standard procedures. 1.5 mL microcentrifuge tube with 0.6 mL sample and 250 μL 0.5 mm glass bead were attached to a horizontal platform on a vortex mixer and agitated vigorously at 2800–3200 rpm for 30 min. Then 7.2 μL lysozyme was added for wall-breaking reaction. 0.3 mL sample was separated into a new 1.5 mL microcentrifuge tube and DNA was extracted using the TIANamp Micro DNA Kit (DP316, TIANGEN BIOTECH) according to the manufacturer’s recommendation. The extracted DNA specimens were used for the construction of DNA library. Then, DNA libraries were constructed through DNA-fragmentation, end-repair, adapter-ligation and PCR amplification. Agilent 2,100 was used for quality control of the DNA libraries. Quality qualified libraries were pooled, DNA Nanoball (DNB) was made and sequenced by MGISEQ-2000 platform (1). Negative control was set as quality control in each test. Low quality reads with short length less than 35 bp were removed in order to maintain high quality sequencing data. By applying Burrows Wheeler Alignment (2), human host sequences were proper computed and deleted, then mapped to the human reference genome (hg19). Rest of the data was filtered by erasing low complexity reads and comparing to the Pathogens metagenomics Database (PMDB), which include viruses, bacteria, fungi and parasites. The classification reference database was extracted from NCBI.^1^ Genome Databases included 1,798 kinds of whole genome sequence resulting from DNA viral taxa, 6,350 types of bacterial genomes or scaffolds, 1,064 cases of fungi in connection with human infection, and 234 parasites related to human diseases (15). 995 reads were mapped to Toxoplasma, while the remaining reads were mapped to common environmental microbes or lab contaminants. Two days later, mNGS unexpectedly detected Toxoplasma gondii, and the mNGS result was confirmed by the specific T. gondii PCR assay on the second day further confirmed this finding. Although the doctors were surprised by this finding, they prescribed Compound Sulfamethoxazole (SMZ) and Azithromycin to treat the patient due to the previously ineffective treatment. After 7 days, the patient’s condition improved significantly, and the patient was discharged with oral medications (Figure 2).

After approximately 6 weeks, the patient exhibited good compliance, expressed satisfaction with the results, and underwent a follow-up examination, resulting in normal PCR results and resolved clinical symptoms.

## Discussion

Toxoplasmosis is a zoonotic disease caused by Toxoplasma gondii infection (2, 17). Cats function as the ultimate hosts, and the pathways for human infection entail the consumption of cysts in undercooked meat, ingestion of oocysts from tainted drinking water, transmission through the placenta, and potentially even the transfer via blood transfusions and organ transplants. In some clinical scenarios, especially in individuals with weakened immune systems (7, 8), such as those diagnosed with acquired immunodeficiency syndrome (AIDS) (18), along with children suffering from leukemia, cerebral toxoplasmosis arises as a nonnegligible complication. Clinical symptoms are multifaceted and varied, with different levels of cerebral symptoms, often presenting as subacute encephalitis, and may manifest as alterations in consciousness, fever, headache, seizures, visual impairment, aphasia, among others (19–21).

According to current findings, tachyzoites of T. gondii are rarely detectable in cerebrospinal fluid and brain tissue by microscopic examination, even though this is considered the gold standard for clinical diagnosis in an ideal situation. The detection of Toxoplasma gondii trophozoites or cysts through microscopic examination using special staining. Immunoperoxidase staining can increase the sensitivity of detection (22), but the positivity rate is low. Worse still, this method is primarily recommended for brain tissue biopsy, which poses a high risk and has limited clinical usefulness. Given that the etiological agent is Toxoplasma gondii, cultivation can be challenging and it may also exhibit insensitivity to other CSF examinations.

In most cases, TE is a consequence of a reactivation therefore IgM are typically negative and IgG are not suitable to distinguish reactivation from latent infection as they persist for several years after an infection (22). However, for the patient, a delayed diagnosis may lead to unbearable consequences. At present, the most commonly used method to confirm TE etiology is CSF Toxoplasma gondii PCR, which has high specificity but variable sensitivities (35–72%) across different PCR systems (23). Specific Toxoplasma PCR testing in blood or CSF is an important component of the first-line diagnostic tests in cases involving suspected cerebral infections in most developed countries, as recommended by international guidelines. However, these assays typically require a prior suspicion of T. gondii infection (24). While clinicians responsible for immunocompetent patients are generally well-informed about toxoplasmosis, identifying Toxoplasma encephalitis in immunocompromised individuals remains challenging (25, 26), especially in the early stages of the disease. This identification primarily relies on a comprehensive evaluation of clinical presentations, imaging assessments, and laboratory investigations. Simultaneously, it is essential to rule out intracranial neoplasms, neurosyphilis, tubercular meningitis, progressive multifocal leukoencephalopathy, cytomegalovirus encephalitis, cryptococcal meningitis, and other diseases. This undoubtedly heightens the complexity of clinical diagnosis and can readily result in delayed or missed diagnoses.

In this case, the patient presented with non-typical clinical manifestations and imaging findings, posing difficulties in precise diagnosis and treatment selection. To avoid delays in critical treatments, healthcare providers implemented a multifaceted antimicrobial regimen. Additionally, the patient underwent a lumbar puncture procedure, and cerebrospinal fluid testing was performed using mNGS technology. The inadvertent detection of Toxoplasma gondii resulted in the ultimate medical diagnosis. This case suggests that mNGS could serve as a valuable diagnostic tool for TE in children with leukemia, consistent with previous research findings (12, 18).

For infections affecting CNS, common clinical detection methods such as imaging and culture exhibit low diagnostic efficiency. PCR and antibody detection techniques require early identification of specific pathogens or a limited set of pathogens, which significantly restricts their use (27). Even when the etiological culture yields a positive result, it will still necessitate a minimum of 2 days, even under the most optimal conditions. Currently, there are no widely accepted antigen, molecular, or nucleic acid-based testing approaches to expedite pathogen identification for CNS infections, leading to a delay in proper treatment. Consequently, diagnosing CNS infections remains challenging due to the lack of rapid and reliable detection methods. Delayed diagnosis and inappropriate therapy may cause permanent damage or death, which is unacceptable. Therefore, clinical doctors require a simple, rapid, and effective method to isolate and detect pathogens.

Culture-based methods remain the standard for etiological diagnosis of infections, but they have disadvantages such as a long turnaround time and low sensitivity in practice. Although molecular assays, such as PCR-based methods, have been established for pathogen detection, their detection is limited to known pathogens listed on the panel or putative pathogens identified by medical professionals. But, mNGS has the capability to identify thousands of pathogens directly from samples in a hypothesis-free and culture-independent manner with a single run. Therefore, this technology can reduce the number of tests required and the time needed for detection (28). Due to these advantages, cerebrospinal fluid second-generation sequencing technology can serve as an auxiliary diagnostic tool for CNS infections. It can rapidly and sensitively detect pathogens, provide information on thousands of pathogens, narrow down the detection range (11), and provide reliable evidence for clinically challenging infections (28–30).

Regarding our patient, the presence of headache, fever, lethargy, and diffuse brain contrast-enhancing lesions were atypical features of Toxoplasma encephalitis (8). The lack of specificity in early laboratory tests and clinical features led to no consensus being reached on the initial clinical diagnosis of the CNS infections. Hence, mNGS of cerebrospinal fluid samples serves as a distinctive role in swiftly and universally detecting potential pathogens in the initial phases of the disease. This method can help guides subsequent clinical diagnosis and treatment, particularly in instances marked by uncertain disease characteristics.

Simultaneously, mNGS confers an additional advantage, rapid and accurate diagnosis is crucial for Chinese doctors, especially due to the tense doctor-patient relationship (31, 32). Additionally, the parents of this child have high treatment expectations. Due to the presence of a severely complicated underlying condition, doctors implemented a combined anti-infective treatment program upon admission to cover all potential pathogens. mNGS platform used in this study had a turnaround time of approximately 24 h from sample receipt. Utilizing this diagnostic technique could achieve a prompt and accurate etiological diagnosis, which could facilitate the optimization of antimicrobial therapy, reduce healthcare costs, improve the doctor-patient relationship, and mitigate drug-related toxicities. This case also indirectly proves the value of applying mNGS sensitivity and specificity in clinical practice.

## Limitation

(1) Owing to the high sensitivity and susceptibility of contamination in mNGS, sterile operation is of utmost importance (33); (2) currently, there are still few large-scale multicenter randomized controlled clinical studies, and the value of mNGS in the diagnosis of infections has not been fully recognized; (3) the interpretation criteria for mNGS results are not completely standardized (34, 35); (4) compared to other specific tests like PCR and antibody testing, mNGS is a valuable early screening tool, only in cases where the direction of early diagnosis is uncertain; (5) this article is a case report and is limited to cases of central nervous system infections.

## Conclusion

For patients presenting with headache, fever, mental disorders, and intracranial abnormal lesions, but with no apparent abnormalities found in routine cerebrospinal fluid examination, it is recommended to promptly conduct second-generation sequencing of cerebrospinal fluid, especially if high-risk factors such as immune deficiency exist. This will establish a basis for clinical diagnosis and treatment, improve early diagnosis and treatment rates, and ultimately reduce disability or mortality resulting from missed, misdiagnosed, or delayed diagnoses.

In general, mNGS has exhibited promise as a valuable tool for diagnosing toxoplasma encephalitis. However, it is crucial to acknowledge that mNGS is still a relatively novel technology, and additional investigations are necessary to comprehensively assess its diagnostic accuracy and clinical usefulness in detecting toxoplasma encephalitis.

## Data availability statement

The raw sequence data reported in this paper have been deposited in the Genome Sequence Archive in National Genomics Data Center, China National Center for Bioinformation/ Beijing Institute of Genomics, Chinese Academy of Sciences, under accession number CRA012435 that are publicly accessible at https://ngdc.cncb.ac.cn/gsa, and further inquiries can be directed to the corresponding authors.

## Ethics statement

The studies involving human participants were reviewed and approved by The Ethical Committee of the First Affiliated Hospital of University of Science and Technology of China (Anhui Provincial Hospital). Written informed consent was obtained from the patient’s parents for the publication of any potentially identifiable images or data included in this article.

## Author contributions

X-GC and H-DM conceived the idea of this article. C-WY and X-FG, contributed to preparation of the manuscript and interpreting patient data. All authors contributed to the review, editing, read, and approved the final manuscript.

## Footnotes

^1^ftp://ftp.ncbi.nlm.nih.gov/genomes/
